# Towards telehealth delivery in pediatric rheumatology practice

**DOI:** 10.1186/s12969-023-00892-x

**Published:** 2023-12-17

**Authors:** Raju Khubchandani, Tadej Avčin, Angelo Ravelli

**Affiliations:** 1 Section of Pediatric Rheumatology, SRCC Childrens Hospital, K Khadye Marg Mumbai, Mumbai, India; 2 Department of Allergology, Rheumatology and Clinical Immunology, Children’s Hospital, University Medical Center Ljubljana, Ljubljana, Slovenia; 3 Department of Pediatrics, Faculty of Medicine, University of Ljubljana, Ljubljana, Slovenia; 4 Direzione Scientifica, IRCCS Istituto Giannina Gaslini and Dipartimento di Neuroscienze, Riabilitazione, Oftalmologia, Genetica e Scienze Materno-Infantili (DINOGMI), Università degli Studi di Genova, Genoa, Italy

**Keywords:** Telemedicine, Telerheumatology, Telepractice, Teleconsultation, Telehealth

## Abstract

**Introduction:**

Much has been written and spoken about telemedicine since about two decades including an article in this journal at the start of the pandemic. It took a global catastrophe to enforce its usage across the world in various medical specialties. Telemedicine however remains unstructured, unregulated and lacks uniformity.

**Discussion:**

This article highlights the practical learnings and opinions of the authors who provided over two thousand video consults and asynchronous telemedicine services through the entire pandemic. It includes lessons learnt from emerging economies where pediatric rheumatologists are scarce. Pediatric rheumatology, which relies heavily on history, musculoskeletal and skin examination is aptly suited to exploit telemedicine in its synchronous and asynchronous forms. Pediatric tele rheumatology could temporarily address the shortage and uneven distribution of specialists in vast parts of the globe, besides serving as a method of triage and shared care with the primary physician. Reduction of direct and indirect costs and family/primary physician education are additional benefits. There also exist challenges for all stakeholders and it is important to address the latter.

**Conclusion:**

The learnings of the pandemic suggest a vital role for telemedicine in the practice of pediatric rheumatology. This is a fertile area for research and consensus building by international and national pediatric societies and issue position statements like some adult bodies already have. The authors speculate a hybrid system of care in the not-so-distant future.

## Introduction

Telemedicine has been defined as “the use of medical information exchanged from one side to another via electronic communications to improve patients clinical health status [1]. Telemedicine encompasses four broad areas namely : TelePpractice (direct physician to patient contact) TeleConsultation (doctors who request consultations with experts for patients under their care), TeleResearch and TeleEducation [1]. TeleMmonitoring may be added to this list. Telemedicine as applied to rheumatology has been christened as Telerheumatology [2]. The educational component itself could be divided into two parts namely professional education of providers (peers or trainees) and further the individual or collective education of the families of afflicted patients [1]. This commentary focuses primarily on the day-to-day delivery of health care to individual children with rheumatologic diseases covering Telepractice, Teleconsultation, Telemonitoring and education of their parents and caregivers. Research and patient surveys not directly impacting individual patient health could be considered an extended product of this exercise. It therefore focusses on doctor to client interactions.Hence forth it shall be referred to as Pediatric Rheumatology Telehealth (**PRT)**. Peer to Peer interactions for training or networking or research are not in the scope of this article.

### Glossary of terms [3]

#### Health Care Provider

The person/s providing pediatric rheumatology care including but not limited to the pediatric rheumatology practitioner, accompanied by his/her Multidisciplinary team. The Hub site or specialist site is the centre/s where this team provides *physical* care. Spoke site (remote site or originating site) is where the child and his caregivers are located.

#### Caregivers

This includes the parents, consented extended family or legal guardians.

#### Presenter or intermediary

A qualified health care person who can serve as a professional liaison and may be physically present at the site of the patient or responsible for gathering the data e.g., physical examination or relevant laboratory results or handling the equipment on behalf of the patient.

#### Synchronous telepractice (ST)

Telepractice that occurs in real time e.g., through video calls, web platforms, telephone calls etc.

#### Asynchronous telepractice (AT)

Transmission of health data (e.g.,history, test results, images, consents, prescriptions, educational material, disease measures via an electronic communication system e.g., email, etc. eConsult is a form of AT.

#### Stakeholders

All persons referred to above *and* professional associations, local health implementation authorities, legal and insurance bodies, and developers of software and hardware that could facilitate PRT.

## Background

The earliest reference of telemedicine in rheumatology is in 1995 in a Texas state prison system [4]. Telemedicine was also first recommended in pediatric rheumatology to address the shortfall in the global workforce [5]. Since that time data and usage has been scant and progress slow though description of its use exists before the COVID 19 pandemic and was reported in this journal [6]. The recent natural calamity of global proportions was a major disruptor of face-to-face patient visits, monitoring, research, teaching activities and meetings. In a backdrop of a limited and skewed work force of pediatric rheumatologists globally and even in resource rich countries [5, 7], sizeable number of children with pediatric musculoskeletal disorders are likely to have received delayed diagnosis, disruption of care or suffered complications due to drug or disease. The burden of this remains unestablished and will be difficult to measure. The only silver lining that emerged was a renewed interest in Telemedicine and there are reports, and real-life experiences of the successful use of pediatric telerheumatology during the pandemic [8]. The American College of Rheumatology has published a position paper on telemedicine [9] and recently the WHO has published a document titled ‘How to plan and conduct telehealth consultations with children and adolescents and their families’ relevant to children” [10]. There is no document from an official body that addresses the merits and challenges of PRT. It is therefore important for pediatric organizations such as Paediatric Rheumatology European Society (PReS), and national pediatric rheumatology organizations /societies to express a viewpoint with respect to PRT. This would support and stimulate constituent members, notably from resource challenged and/or underserved countries to further enhance and incorporate PRT in their workplace. This document represents a viewpoint of the authors in their individual capacities on the subject.

## General comments

A. Most literature on telemedicine, especially with respect to adult or pediatric rheumatology comes from resourceful countries [3]. In the wake of the pandemic, several countries such as India [11] passed emergency legislations to legitimize telemedicine shortly after the lockdowns, but in many countries laws related to telemedicine are no different from those existing for regular health care delivery e.g. Australia or continue to evolve e.g. Germany or even vary by geography at state levels e.g. USA [12]. This hugely potential resource thus remains non- uniformly and locally governed. It is now time to take stock of the learnings of the last two years and pivot systems of health care delivery in areas and specialties where applicable. In fact, advantage needs to be taken of the progressive increase in access to smart phones and internet in resource challenged countries. Stakeholders should leverage this to reach the distant and / or disadvantaged using the experiences of the last two years to scale up and generate data that can highlight their triumphs and tribulations with PRT.
B. This document could help all colleagues and stakeholders pool their ideas under the auspices of professional associations such as PReS / EULAR. This could lead to a ‘points to consider’ document which addresses medical, legal, logistically easy and secure framework and uniform international standards to incorporate PRT in the delivery of care.

The forthcoming sections address the strengths or opportunities and weaknesses or threats from the perspective of all stakeholders and are discussed in each section not necessarily in their order of importance.

## Patient

### Strengths and opportunities

A. The importance of a first in- person meeting for rapport building cannot be understated and lack of physical contact has been cited as a barrier to telemedicine [13]. However, in exceptional situations such as pandemics, wars, natural calamities PRT could provide a viable alternative. Some specific chronic rheumatologic conditions where physical examination is less relevant can be followed up via PRT alternating with physical visits (suggested list Table 1).The central column in this table lists some conditions which can be diagnosed with confidence on the screen as per the author’s experience while others can be suspected strongly and /or moved for rapid treatment to specialist centers. Idiopathic inflammatory myopathies is an example of a condition that has been the target of such studies [14] though the list needs to be extended based on evidence.
B. Pediatric musculoskeletal disorders rely dominantly on bedside evaluation. History taking is possible via ST or AT, with previous records, reports, images etc. being transmitted ahead of ST by email or similar. There is now a video version of pediatric Gait Arms Legs and Spine (pGALS) which instructs joint examination [15]. In the experience of one of the authors (RPK) during follow up physical visits some parents could be taught specific signs involving passive joint examination relevant to their child or these could be demonstrated via images or videos in the ‘share screen’ mode of video conferencing software [8].
C. Gait and movement disorders can be observed on ST or transmitted as video clips via AT.
D. The skin provides several clues to the pediatric rheumatologist. Tele dermatology is well developed and amongst the earliest beneficiaries of Telemedicine [16]. Examination of the conjunctiva, palate and even nails (using the phone camera) are possible.
E. The caregivers or the intermediary can record anthropometry, and blood pressure with home use instruments. In undiagnosed cases, especially, at the first consult, an intermediary can be used to record auscultatory findings until remote real time internet enabled auscultation gains further traction. There is an infrequent requirement of instruments such as stethoscopes, otoscopes etc. for evaluation of patients with musculoskeletal complaints. If required, the services of the intermediary or presenter can be utilized. For various rheumatological diseases it is important to create an essential or sufficient component of data required for follow up visits as has been elegantly listed [7].
F. Several pediatric rheumatology scales of activity e.g. CMAS or damage e.g. SLEDAI or quality of life e.g. KIDSCREEN are possible to evaluate through ST. Disease activity and QoL questionnaires can be transmitted via AT for use during the next online or in-person meeting [17].
G. Prescriptions can also be transmitted online. The Hub team can transfer Protocols for intravenous medications to the presenter or intermediary.
H. Most children attending rheumatology visits on follow up are immunocompromised due to their drugs. Telemedicine could reduce the risk of cross infections from a busy multispecialty hospital outpatient department with long wait times [18].

**Table 1:** Suggestions for some potential situations for Pediatric Rheumatology Telehealth (list illustrative)

Disease/Condition/Signs	Triage(T)/Suspicion(S)/Diagnosis(D)	Hybrid meetings with monitoring and treatment
Macrophage activation syndrome	T, S, D	Feasible
Acute infections e.g., osteomyelitis, septic arthritis	T, S	Feasible
Kawasaki Disease	T, S, D	Feasible
IgA vasculitis	D	Feasible
Juvenile Dermatomyositis	S, D	Feasible(including Childhood Myositis Assessment Score)
Systemic Lupus Erythematosus (SLE)	S, D	Feasible (including SLE Disease Activity Index)
Localized Scleroderma (most forms)	D	Feasible
Systemic sclerosis	D	Feasible
Psoriatic arthritis	S, D	Feasible /Possible
Chronic Nonbacterial Osteitis		Feasibleasible / Possible
Gait and movement disorders	S	
Early onset hereditary autoinflammatory diseases	T, S	Few feasible e.g., DADA 2 in remission.

### Weaknesses and threats [13]

A. Examination of pre-school children, toddlers and infants on a screen may be difficult and incomplete. Similarly examination of children with significant physical or mental handicap on screen can be difficult [19].
B. Examination of the swimsuit area in females or genitals in males with images transmitted on the internet is inappropriate and local cultural beliefs may also hinder exposure of other body parts.
C. Non-verbal cues and body language are difficult to evaluate during ST. One- on- One dialogue with an older child or adolescent without parental presence may be difficult to achieve.

## Care provider

### Strengths and opportunities

A. PRT can allow flexibility of number of caregivers accompanying the patient for a physical visit allowing other interested and consented caregivers to join the physical meeting online [3].
B. In several resource challenged countries there is migration of key earners to other geographies, leaving families behind. PRT offers an opportunity to counsel all members of the family or extended family simultaneously even if they are not under the same roof at the time of the consult.
C. PRT can be invaluable in children with chronic disease where social challenges e.g., single parent / affected sib / orphanages / remand homes add a ‘second hit’.
D. The savings of direct and indirect costs and time to the families could be significant.

### Weaknesses and threats

A. In resource challenged settings parents may feel embarrassed to reveal their home environment.
B. In resource challenged countries the smart phone is the only internet connected device available and are often limited to one per family.
C. Parent satisfaction with consults in non-emergency situations needs to be studied [20].
D. In patients in apparent remission there could be a development of quiet complacency thus avoiding or postponing future physical visits. This can delay identification of low-grade disease activity or flares.

## Health care professional

### Strengths and Opportunities

A. As per the WHO definition most chronic pediatric rheumatologic / musculoskeletal / genetic conditions are classified as rare or ultra-rare [21]. Awareness about these diseases is rather low even in resourced settings. PRT offers an opportunity to experts to be able to identify suspicious cases and call for a face-to-face visit. PRT can thus expand the geographic limits of outreach clinics from Hubs within countries, thereby attempting to cover at least a fraction of the gap in the availability of trained and qualified pediatric rheumatologists.
B. Current teaching of pediatric rheumatology at the undergraduate and post graduate levels is scant. Thus, primary care practitioners need assistance in diagnosis and monitoring of children with musculoskeletal ailments. PRT can encourage the engagement of shared care with the intermediary thus progressively increasing awareness of the nuances of longitudinal care of the diseases we treat.
C. Emergencies in pediatric rheumatology though infrequent, need rapid identification and triage. Shared care with rapid availability of the Hub to the primary care practitioner can address this issue.
D. PRT can be used for interdisciplinary consults (e.g., transition clinics, ophthalmology, dermatology, reviewing biopsy results with pathologists, reviewing images with radiologists etc.). This saves a considerable amount of time for the patient who is often attempting to coordinate multiple specialists who may not be at the same hospital or even city. Such consults can be scheduled at off peak hours.
E. PRT allows the option of the consultants or decision makers at the Hub being available to their patients directly or via their team even during periods of travel or leave.
F. Accessibility, Availability and Affordability can be challenges for patients with rheumatologic illnesses to specialist rehabilitative specialists e.g., physiotherapists, mental health counsellors and dieticians. In resource challenged settings patients and their parents often prioritize drug therapy over these aspects of care. Telephysiotherapy has immense potential as an allied offering [22].
G. The nurse practitioner can contribute by demonstrating subcutaneous injection techniques, administer questionnaires, send educational material, address prescription clarifications/errors and check on drug doses and LASA (look alike sound alike) drugs. Above all she (or he) serves as the point of first contact.

### Weaknesses and threats [23]

A. Screen fatigue can be a source of stress for the caregivers.
B. In attempting a hybrid day, time management poses challenges. Time overruns from the physical visits have the potential to disrupt or delay the start of virtual consults leaving families in the virtual waiting room uncertain and anxious with delays in their onward plans for the day. Switching from one patient to the next could also consume time as the next entrant has logged off, needs device and lighting adjustment etc. This reduces time efficiency of the Hub.
C. The Hub would need to address payment structure /mode, account keeping, and other financial issues.

## Social / community / national ecosystem

### Strengths and opportunities

A. PRT can open a whole new vista and international dialogue for creating a framework for international consultations with chosen experts in their very narrow but path-breaking areas of work.
B. Children with pediatric rheumatologic illnesses contribute significantly to chronic health care burden in a country. Macroeconomic benefits of managing such children using telemedicine, though not yet quantified, could be significant.

### Weaknesses and threats

A. Technical challenges (software, hardware, internet speed, ability to understand instructions, multilingualism) may limit the quality of the consult and thus reduce patient satisfaction.
B. All stakeholders need to address record keeping, integration of PRT into the electronic medical records, maintenance of data privacy, local and international legislation e.g., EU and payment or insurance mechanisms.
C. Appropriate instructions, consents, limitations, and disclaimers translated in local languages clearly understood by the patients and their caregivers need to be communicated prior to the consult.

## Conclusion

The recent pandemic has revived interest in telemedicine and preliminary reports and experiences have shown that pediatric rheumatology as a branch could be a big beneficiary.

There is an urgent need to build evidence around the practice, identify areas of research and development (Table 2).

**Table 2:** Some potential areas of research and development for pediatric telehealth delivery

Research	Validation of diagnoses and decisions for various conditions
	Patient and caregiver satisfaction
	Cost efficacy
	Optimal device/s
	Validation of QoL indices/activity and damage indices normally used for in-person visits,
Development	Software for optimal physician and patient experience, including privacy
	A legal framework to protect both physician and patient
	A legal framework to allow international consults.
	Record keeping to be integrated into existing EMR
	Equipment for remote examination e.g., stethoscopes.

It is therefore an opportune moment for national societies and international bodies such as PReS, EULAR and the Global Health Task Force to seize the opportunity and lay an agenda for rapid action before losing the momentum created by the pandemic. Bringing all stakeholders under one umbrella to address the strengths opportunities, threats and weaknesses is the need of the hour. Indeed as was written in this journal [24] a hybrid system seems to be emerging as a method of health care delivery with pediatric rheumatology as a major beneficiary. It has taken a pandemic to accelerate a resource available for two decades.

Abbreviations:

PRT-Pediatric Rheumatology Teleheaslth, ST-Synchronous Telepractice, AT-Asynchronous Telepractice, pGALS-pediatric Gait Arms Legs Spine.

## Data Availability

Not applicable.
